# Long-term outcomes of the classic Konno–Rastan procedure in paediatric and adult patients: impact of aortic annulus size on patient outcomes

**DOI:** 10.1093/icvts/ivad151

**Published:** 2023-09-04

**Authors:** Ahmad Ali Amirghofran, Maryam Salimi, Hooman Kamran, Hamed Bazrafshan, Mohammad Rafati Navaei, Ali Shokrollahi, Elahe Nirooei, Mohammadreza Edraki, Hamid Amoozgar, Gholamhossein Ajami, Hamid Arabi

**Affiliations:** Cardiac Surgery Department, Shiraz University of Medical Sciences, Shiraz, Iran; Student Research Committee, Shiraz University of Medical Sciences, Shiraz, Iran; Student Research Committee, Shiraz University of Medical Sciences, Shiraz, Iran; Cardiac Surgery Department, Shiraz University of Medical Sciences, Shiraz, Iran; Cardiac Surgery Department, Shiraz University of Medical Sciences, Shiraz, Iran; Cardiac Surgery Department, Shiraz University of Medical Sciences, Shiraz, Iran; Cardiac Surgery Department, Shiraz University of Medical Sciences, Shiraz, Iran; Pediatric Cardiology Department, Shiraz University of Medical Sciences, Shiraz, Iran; Pediatric Cardiology Department, Shiraz University of Medical Sciences, Shiraz, Iran; Pediatric Cardiology Department, Shiraz University of Medical Sciences, Shiraz, Iran; Cardiac Surgery Department, Shiraz University of Medical Sciences, Shiraz, Iran

**Keywords:** Konno–Rastan procedure, Prosthetic valve, Aortic stenosis, Aortic regurgitation, Patient–prosthesis mismatch

## Abstract

**OBJECTIVES:**

The classic Konno–Rastan procedure may yield different outcomes regarding aortic annulus diameters ≤15 mm and larger. Focusing on the effect of the diameter of the aortic annulus, we described the long-term outcomes of our patients.

**METHODS:**

The outcomes of paediatric and adult patients who underwent surgery from 2000 to 2021 were studied retrospectively. The patient population was divided into 2 groups with aortic annulus diameters ≤15 mm and >15, and the outcomes were compared between the 2 groups.

**RESULTS:**

A total of 48 patients, with a mean age of 12.24 ± 9.42 years (2–53 years) and a median follow-up duration of 8 years (7 months to 20 years) with an IQR of 5.5, were enrolled. The mean peak instantaneous pressure gradient was 78.97 ± 25.29 mmHg, which decreased to 21.43 mmHg (*P*-value = 0.012). The maximum left ventricular outflow tract gradient at the last follow-up was 28.21 mmHg, with the exception of 1 case with a gradient of 68.45 mmHg. The mean diameter of the aortic annulus was 15.34 ± 3.87 mm (8–23 mm), and the mean prosthetic valve size was 20.31 mm, which was 5 mm (33%) larger than the native annulus diameter. The overall mortality rate was 6.3%, with 1 death in the hospital and 2 in the first year after the surgery. The major complication rate, including mortality, heart block and reintervention, was higher in patients with ≤15 mm annulus (*P*-value = 0.028.) However, there was no difference between the 2 groups in follow-up. Four (8%) late cardiac reoperations were performed, none of which were related to our surgeries.

**CONCLUSIONS:**

Kono–Rastan surgery for patients with aortic valve diameter of ≤15 mm can be performed with acceptable long-term outcomes.

**Figure:**
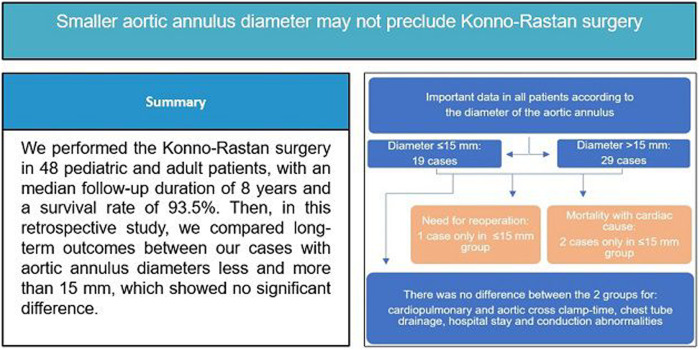

## BACKGROUND

The prosthesis–patient mismatch (PPM), which was first described by Rahimtoola [1], is a condition in which the effective orifice area of the prosthetic valve is smaller than expected relative to the patient’s body size [2]. It is associated with unacceptably high transvalvar pressure gradients, which lead to poor haemodynamics, reduced postoperative recovery and shorter long-term survival [3]. Aortic root enlargement is one of the methods to prevent PPM in the case of a small aortic annulus by placing a prosthesis of an appropriate size [4].

The Konno–Rastan procedure, also known as anterior aortoventriculoplasty, is a useful technique for aortic valve replacement with annulus enlargement. This procedure, which was first described by Rastan and Koncz [5] and also Konno *et al.* [6] in 1976 for tunnel-like subaortic stenosis, has been used to relieve subvalvar, valvar and supravalvar stenosis [7]. By using this procedure, it is possible to eliminate obstruction and stenosis from the left ventricular outflow tract (LVOT) up to the ascending aorta and replace the valve with a prosthesis that is significantly larger than the existing annulus, even in small children. Nevertheless, the need for anticoagulation, the risk of valve thrombosis or degeneration and the possibility of late PPM with advancing age are among the disadvantages of this procedure that need to be weighed against its advantages in long-term follow-ups [7, 8].

The present study was conducted to evaluate the outcome of patients with the Konno–Rastan procedure in our centre over a 20-year period. Furthermore, we examined the effect of annulus size on the rate of complications by comparing the results regarding native annulus diameters below and above 15 mm. This cut-off line was chosen right in the middle of the aortic annulus size spectrum to divide the cohort into 2 groups with small annulus (>15 mm) and very small annulus (≤15 mm) to observe if the more demanding procedure in the very small annulus group directly affects the outcome.

## MATERIALS AND METHODS

### Ethical statement

All the procedures performed in this retrospective study followed the ethical standards of Shiraz University of Medical Sciences research committee and the 1964 Helsinki declaration and its later amendments or comparable ethical standards. The surgical procedure was explained to the patients or guardians, and the informed consent form was completed. The ethics committee code number of this study is IR.sums.med.rec.1400.218.

In this retrospective study, all consecutive paediatric and adult patients, who underwent the Konno–Rastan operation in 2 hospitals affiliated with Shiraz University of Medical Sciences, Shiraz, Iran from April 2000 to September 2021, were enrolled, and their surgical outcomes were examined.

Before starting the study, we expected that patients with a larger annulus would have a significantly better outcome than patients with a smaller annulus.

The classic Konno–Rastan procedure was performed by aortic root enlargement with anterior aortoventriculoplasty and mechanical prosthetic valve prothesis replacement of the aortic valve except for 2 patients with biological valve.

The inclusion criteria, in compliance with the American Heart Association guidelines, were as follows: a small aortic valve annulus, with or without narrowing of the subvalvar area, with a peak instantaneous gradient ≥60 mmHg or a mean pressure gradient ≥40 mmHg or an aortic valve area ≤0.5 cm^2^/m^2^; cases with a peak instantaneous gradient ≥50 mmHg or an aortic valve area of 0.5–0.8 cm^2^/m^2^ in the presence of symptoms, such as angina, syncope and electrocardiographic changes or in cases planning for pregnancy; and cases with a peak instantaneous gradient ≥35 mmHg or an aortic valve area of 0.8–1.5 cm^2^/m^2^, with more than moderate aortic regurgitation due to valve deformity or previous ballooning [9, 10].

Patients with an atrioventricular block or a cardiac conduction abnormality before the surgery and those undergoing modified Konno and Ross–Konno surgeries were not included in this study.

The demographic and hospital data of the patients, including their weight, sex, body surface area, cardiopulmonary bypass time, aortic cross-clamp time, surgery duration, chest tube drainage, intensive care unit stay and total hospital stay, were derived from their hospital charts.

Furthermore, changes in echocardiographic parameters were assessed prior to surgery and during subsequent follow-up. Transthoracic echocardiography was performed before and early after the operation, and the patients were followed-up at regular 6- to 12-month intervals, using the M-mode, two-dimensional and Doppler methods according to the guidelines. The electrocardiograms of the patients were compared before and after the operation for any conduction alterations. These variables were compared between patients with aortic annulus diameters of ≤15 and >15 mm to determine any significant differences.

To evaluate the impact of annular size on post operative complications, we evaluated the early (in-hospital) and late major complications and compared them in the 2 groups.

### Surgical technique

**Figure 1:**
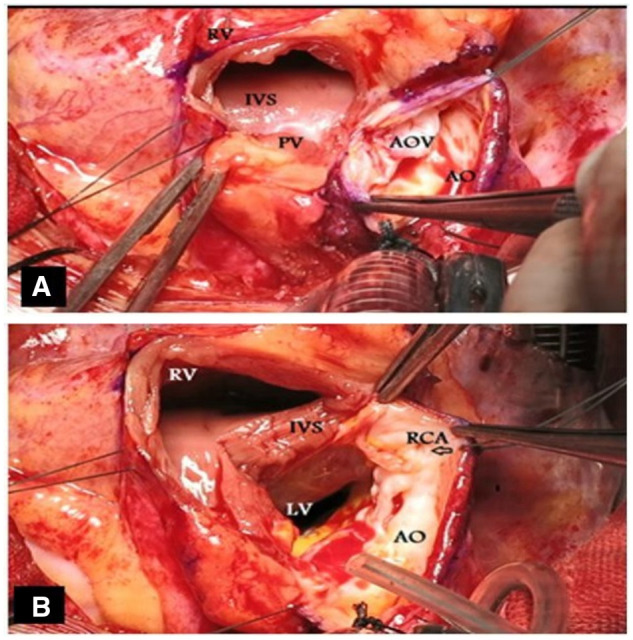
(A) The longitudinal incision on the lines meets in the middle part between the right coronary artery orifice and the pulmonary valve annulus. (B) extension of the aortic incision towards the septum near the commissure between the right and left coronary cusps.

All operations were performed through a median sternotomy and cardiopulmonary bypass with cardioplegic arrest. The details of the operation have been described by Konno and Rastan. In brief, the lines of the longitudinal incision were marked on the aorta and the right ventricular wall (video 1). The lines met in the middle part between the right coronary artery (RCA) orifice and the pulmonary valve annulus, while keeping a safe distance from adjacent anatomical structures, especially from the RCA orifice. A longitudinal incision was made on the aorta, and the anatomy of the valve was examined. If replacement of the valve with at least a 19-mm prosthetic valve seemed impossible, right ventriculotomy was performed, which continued towards the septum, a few millimetres apart from the pulmonary valve (Fig. 1A).

The aortic incision continued towards the septum near the commissure between the right and left coronary cusps. At this point, the annulus was cut a few millimetres apart from the RCA origin, and the incision was advanced into the interventricular septum. Additionally, an edge myectomy of the septum was performed to decrease the septal thickness and tunnel narrowing (Fig. 1B). The aortic valve cusps and fibrotic tissue were then excised, and the opened annulus was evaluated by an aortic valve sizer.

In the procedures, we tried to avoid a prosthetic valve size more than twice the size of the opened annulus. In other words, at least half of the new valve ring should be sutured to the opened native annulus, and the anterior half should be sutured to a Dacron patch. The ellipsoid-shaped Dacron patch was trimmed, with the width of its middle part equal to the length of annulus enlargement and the length from the aortotomy inferior corners to the septostomy end corners.

**Figure 2:**
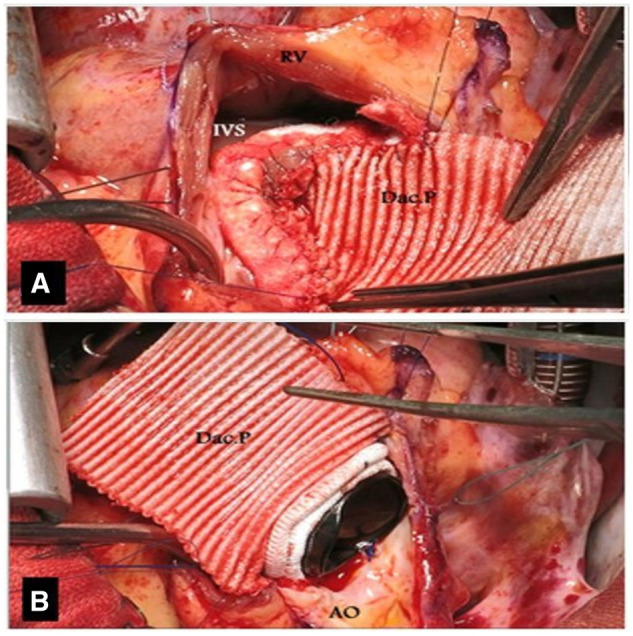
(A) The Dacron patch is trimmed so that the width of its middle part is equal to the circumference of the annulus that is to be enlarged. (B) The length of the Dacron patch is extended from the aortotomy to septostomy end corners.

**Figure 3:**
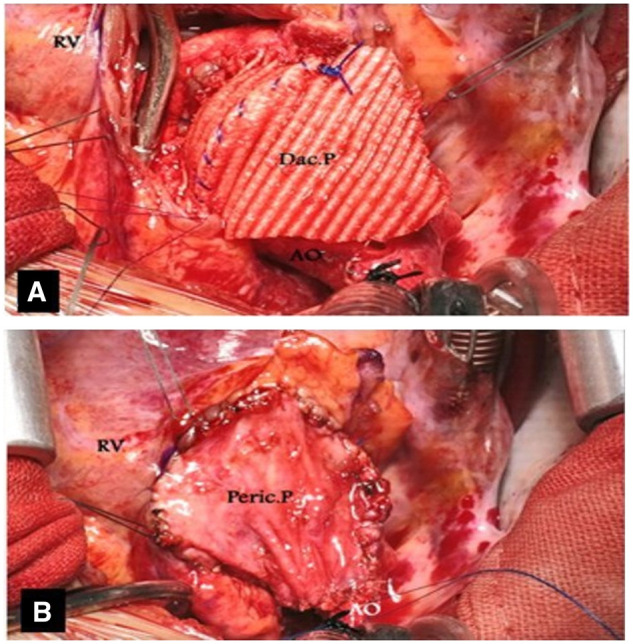
(A) A Dacron patch is continuously sutured to the anterior half of the prosthetic valve from the outside. (B) A pericardial patch is used to close the right ventriculotomy.

The Dacron patch was sutured continuously to the septal ends and ended at the annulus on both sides. This suture line was reinforced by Teflon tapes over the septal muscle (Fig. 2A). The aortic prosthetic valve was then sutured continuously to the opened native annulus posteriorly (Fig. 2B). Next, the Dacron patch was continuously sutured to the anterior half of the prosthetic valve from the outside. The remaining distal part of the Dacron patch was used to close the aortotomy incision (Fig. 3A and B). The right ventricular opening is commonly closed by a pericardial patch to eliminate the risk of right ventricular outflow tract narrowing due to bulging of the prosthetic valve and septal patch; this pericardial patch may or may not cover the Dacron patch of the ascending aorta.

### Statistical analysis

The patients’ demographic, clinical and paraclinical data were analysed in SPSS version 22.0. Independent sample *t-*test was used to compare the parametric data determined by the mean and standard deviation. The Mann–Whitney *U*-test was used to compare nonparametric data using median and interquartile range, p25 and p75 (that are addressed in parentheses).

Also, Wilcoxon rank test were used to compare the values before and after the procedures, and Chi-squared test to compare categorical variables, and Kaplan–Meier and life table for survival analysis. The missing data were excluded from the analysis. *P*-value <0.05 was considered statistically significant.

## RESULTS

A total of 48 patients were included in this study, with a mean age of 12.24 ± 9.42 years (range, 2–53 years) and a median follow-up duration of 8.0 (5.50; 11.00) years, from 7 months to 20 years and a follow-up index of 1.00 (0.00).

Nine patients were younger than 5 years, 12 were between 5 and 10 years old, 13 were between 10 and 15 years old and 14 cases were older than 15 years old. The youngest patient was 2 years old with a weight of 11 kg, and the oldest was 53 years old with a weight of 75 kg.

The study population consisted of 41.4% male and 58.6% female patients. The aortic annulus was 15 mm or smaller in 19 cases, and larger than 15 mm in 29 cases.

The demographic and follow-up data of the 2 groups are presented in Table 1.

**Table 1:** The demographic data of patients with aortic valve annulus diameters ≤15 and >15 mm

Variables	Annulus diameter		*P*-value
	≤15 mm	>15 mm	
	*N* = 19	*N* = 29	
Age at surgery (years)	7.00 (8.13: 4.87; 13)	12.00 (6.00: 9; 15)	0.069*
Weight at surgery (kg)	23.28 ± 11.15	49.93 ± 3.88	0.007**

* Mann–Whitney test, median (IQR: p25; p75).

** Independent sample *T*-test, mean ± standard deviation.

IQR: interquartile range.

There was a total of 46 previous cardiac operations or balloon aortic valvuloplasty procedures had been performed before the Konno–Rastan operation, the details of which are shown in Table 2.

**Table 2:** Important cardiac and non-cardiac operations and percutaneous interventions performed before, during and after the Konno–Rastan procedure for all patients

Variable	Number	Annulus diameter	
		≤15	>15
Previous cardiac surgeries or percutaneous interventions			
No	12	3	9
One surgery	18	7	11
Two surgeries	6	2	4
Three surgeries	2	1	1
Balloon aortic valvuloplasty	10	6	4
Previous cardiac operations:			
Aortic commissurotomy with or without web resection	25	7	18
Modified Konno	2	1	1
Mitral valve repair	2	2	0
Mitral valve replacement	2	0	2
Aortic valve replacement (small diameter)	2	2	0
VSD closure	1	1	0
Homograft for pulmonary atresia	1	0	1
Aneurysm of sinus of Valsalva	1	0	1
Liver transplantation	3	2	1
Combined operations with Konno–Rastan			
Mitral valve repair	5	1	4
Mitral valve replacement	4	1	3
Tricuspid valve repair	2	1	1
CABG	4	1	3
Postoperative operations			
CABG	1	0	1
Liver transplantation	3	1	2
Mitral valve replacement	3	1	1
VSD closure	1	0	1

CABG: coronary artery bypass grafting; VSD: ventricular septal defect.

Fifteen concomitant surgeries were performed for 9 cases mainly on the mitral valve, including a 22-year-old patient with familial hypercholesterolaemia and previous liver transplantation, who underwent coronary artery bypass grafting (4 grafts), mitral valve replacement, tricuspid valve repair and the Konno–Rastan operation simultaneously.

Six of our patients were known cases of familial hypercholesterolaemia, and liver transplantation was performed before or after the Konno–Rastan procedure (Table 2).

According to Table 3, which presents the preoperative, operative and early postoperative data, there were no significant differences regarding the echocardiographic and surgical findings between the 2 groups.

**Table 3:** The preoperative, operative and early postoperative echocardiographic data of the total study population as well as subgroups with 2 annular diameters

Variables	Annulus diameter			*P*-value of the 2 groups
	All patients	≤15-mm group	>15-mm group	
	*N *= 48 (mean ± SD)	*N* = 19	*N* = 29	
LVOT PG before Konno (mmHg)	78.97 ± 25.29	75.80 ± 27.49	80.30 ± 25.14	0.606**
CPB time (min)	102.05 ± 25.36	976 ± 14.04	108.26 ± 32.58	0.271**
ACC time (min)	86.52 ± 27.53	84.38 ± 18.67	89.20 ± 26.42	0.579**
Chest tube drainage (cc/kg)	14.32 ± 9.97	12.41 (12.85: 5.48; 18.33)	13.24 (6.17: 10.95; 17.12)	0.777*
ICU stay (days)	3.83 ± 4.54	3.00 (1.00: 2; 3)	3.00 (3.00: 2; 5)	0.219*
Hospital stay (days)	7.62 ± 5.46	6.00 (1.50: 5.50; 7)	6.00 (3.00: 5; 8)	0.981*

* Mann–Whitney test, median (IQR: p25: p75).

** Independent sample *T*-test, mean ± standard deviation.

ACC: aorta cross-clamp; CPB: cardiopulmonary bypass; ICU: intensive care unit; IQR: interquartile range; LVOT: left ventricular outflow tract; PG: pressure gradient; SD: standard deviation.

While the majority of patients suffered from moderate or severe aortic valvar or subvalvar stenosis, the prominent pathology was severe aortic valve regurgitation in some patients, mainly after balloon valvuloplasty for valve stenosis. The mean size of prosthetic valves was 20.31 mm (range, 19–23 mm), and comparing with the mean preoperative annulus size 15.34 ± 3.87 mm (range: 8–23 mm) with the Wilcoxon rank test showed that this procedure significantly increased the annular size by 33% (5 mm) (*P*-value = 0.0001). Our youngest 2-year-old patient had the smallest preoperative valve diameter of 8 mm.

All patients with an annulus diameter of ≤15 mm had a 19-mm prosthetic valve, while most patients with an annulus of >15 mm received valves with a diameter of 21 or 23 mm (*P*-value = 0.003).

The overall peak left ventricular-aortic pressure gradient dropped significantly from a peak instantaneous gradient of 78.01–21.43 mmHg after surgery (*P*-value = 0.012), and the overall mean gradient decreased from 54.12 to 13.6.75 mmHg. The mean maximum LVOT gradient at the last follow-up was 28.21 mmHg. There is 1 patient who has a high peak gradient of 68 mmHg.

None of the patients with standard anticoagulation therapy experienced valve thrombosis, thromboembolism, endocarditis or other valve-related problems.

Re-exploration for bleeding was performed for 4 cases (8%), 2 in each group. Five late cardiac reoperations were done, none of which were related to the Konno–Rastan procedure (Table 2). Reoperation was performed for reasons unrelated to Konno surgery, including 1 case for coronary artery bypass graft, 3 cases for mitral valve replacement and 1 case for missed apical ventricular septal defect closure. Three patients also underwent liver transplantation due to hypercholesterolaemia. Regular postoperative functional evaluations showed satisfactory results in the rest of the patients.

The incidence of early and late major complications related to Konno–Rastan surgery was compared using a Chi-square test (Table 4). Three patients in the group with an annulus diameter <15 mm (15.78%) developed major complications in the hospital, with significant statistical differences (*P*-value = 0.028). Two patients in the same group (10.52%) had late major complications without any significant difference (*P*-value = 0.077).

**Table 4:** Early (in-hospital) and late major complications among all patients

Variables		Annulus diameter, *n* (%)			*P*-value (total proportion)
		All patients	≤15 mm group	>15 mm group	
		*N* = 48	*N* = 19	*N* = 29	
Early after surgeries	Need for reoperation	0	0	0	0.028
	Mortality related to the surgery	1 (2.08%)	1 (5.26%)	0	
	Atrioventricular block requiring a permanent pacemaker	2 (4.16%)	2 (10.52%)	0	
	Total	3 (6.24%)	3 (15.78%)	0	
Late after surgeries	Need for reoperation	1 (2.08%)	1 (5.26%)	0	0.077
	Mortality related to the surgery	1 (2.08%)	1 (5.26%)	0	
	Atrioventricular block requiring a permanent pacemaker	0	0	0	
	Total	2 (4.16%)	2 (10.52%)	0	

There was no reoperation for the aortic valve stenosis or regurgitation in either group; however, 1 patient with annulus ≤15 mm who had a pressure gradient 68.45 mmHg across a 19-mm prosthetic valve has been enlisted for reoperation, but the parents’ refused surgery.

The hospital mortality rate was estimated at 2.1% (*n* = 1), and the later mortality rate was 4.2% (*n* = 2). The patient who died in the hospital was a 3-year-old girl who had undergone 2 previous surgeries, including a mitral valve repair, followed by a biological mitral valve replacement with rapid degeneration and severe tunnel-like LVOT obstruction. The Konno operation and mitral valve replacement using a prosthetic valve were performed in the third surgery, and the patient died due to severe right ventricular failure on the second postoperative day.

There were also 2 late mortalities, both 5-year-old boys, one of whom had a history of liver transplantation. This patient had abnormal coronary anatomy, with the RCA originating from the posterior aspect of the ascending aorta to the right aspect of the ascending aorta stretched after the Konno procedure, which was managed by a right internal mammary artery graft on the RCA. The patient died of liver transplantation complications 4 months after the transplantation. The cause of late death in the other patient, who died 8 months after the operation, was unclear, although it was attributed to sudden arrhythmia. Overall, the 20-year cumulative survival rate was 93.7% procedure based on Kaplan–Meier curve.

The patients’ conduction abnormalities are presented in Table 5, showing no significant differences between patients with annulus diameters of ≤15 and >15 mm (*P*-value = 0.344). There were only 2 patients who required a permanent pacemaker. One patient was a 5-year-old boy with an abnormal leftward rotation of the RCA, who showed RCA malperfusion after surgery; it was managed by right internal mammary artery grafting, although a high-grade second-degree atrioventricular block was maintained. This patient had previous liver transplantation and died several months after the surgery. The other patient was a 2-year-old boy with an annulus size of 8 mm, who required a pacemaker after the Konno–Rastan operation and mitral valve repair. Both patients requiring a pacemaker were in the group with annulus ≤15 mm.

**Table 5:** Conduction abnormalities in patients with aortic annulus diameters ≤15 and >15 mm

Conduction abnormalities	Annulus diameter	
	≤15 mm *N* = 19	≥15 mm *N* = 29
No abnormality	13	17
Left bundle branch block	6	8
Right bundle branch block	0	2
High-grade second-degree atrioventricular block	1	0
Complete atrioventricular block	1	0

## DISCUSSION

The Konno–Rastan technique for the treatment of LVOT obstruction with complex stenotic stenosis has advantages and drawbacks that should be investigated, especially in patients with a small aortic valve annulus [2]. In this study, we described our experiences of performing this procedure with the long-term follow-up of our patients. The results indicated acceptable outcomes for patients in a wide age range (2–53 years).

The 30-day mortality rate of our cases was 2.1%, and the survival rate at follow-up was 93.5%, with a mean follow-up duration of 9.5 years; while some studies have reported up to 10 and 30% for early and late mortality respectively [11, 12]. Furthermore, there were no significant surgical complications during the follow-up. Although there is a risk of prosthetic valve outgrowth, especially in young children in this surgery, there were no late reoperations related to the Konno–Rastan surgery, despite the minimum age of 2 years in our patients.

Another objective of this study was to evaluate the impact of preoperative aortic annulus size on the patients' operative outcomes, since the use of significantly larger prosthetic valves, especially in smaller children, may be a cause of concern. Interestingly, no significant differences were found between patients with aortic annulus diameters ≤15 and >15 mm regarding the perioperative data and hospitalization course. It should be noted that searching in the literature, we did not find any study to compare these data in groups with different aortic annulus diameters.

One of the major complications following this surgery is the complete atrioventricular block, requiring pacemaker [13]. In some studies, the incidence of heart block requiring pacemaker insertion was >12.5% [12, 13]. However, in the current study, only 2 cases (4.2%) developed 3rd degree, and high-grade second-degree atrioventricular blocks, who were 2 and 5 years old, respectively. Although there was no statistically significant difference in the prevalence of different types of heart block between the 2 groups in our study, the aortic annulus diameter might be an influential factor, as both patients were younger than 5 years old and had annular diameter smaller than 15 mm. Disturbance in electrical conduction may occur due to direct damage to the conducting pathways by the incision itself or the suture line, the pressure of the oversized rigid prosthetic valve ring on the conduction system, or less possibly, inadequate perfusion of the RCA or its branches due to dislocation or angulation [14]. These mechanisms may explain the possible higher prevalence of heart blocks in younger patients with smaller hearts.

The low number of complete heart blocks in the present study may be related to our effort to make the incision on the annulus and septum far to the left, away from the RCA ostium and the conduction system and as close as possible to the right/left commissure and the pulmonic valve.

We also tried not to use a prosthetic valve larger than twice the native annulus diameter. Therefore, the opened native annulus occupies almost more than half of the prosthetic valve circumference, and the pressure effect on the surrounding structures, including the RCA ostium and the conduction system, can be minimized.

The prosthetic valve-related complications, such as difficult anticoagulation control especially in children, infection and thrombus and pannus formation, may complicate the patients’ condition [4]. Also, the prosthetic valve may be outgrown in children and cause PPM if the aortic root enlargement and the inserted valve size are inadequate [7]. In our study, the minimum prosthetic valve size was 19 mm, and we tried to use valve brands with a larger effective orifice area. No patient underwent reoperation for PPM, and only 1 patient had an unacceptably high gradient requiring surgery as noted in the Result section.

Additionally, according to a study by Suri *et al.* [7], pulmonary complications might occur following surgery due to damage to the pulmonary valve, injury to the muscle section or distortion of the LVOT by the right ventricular patch. They reported that 3 patients (4.9%) required reoperation because of this condition. However, we did not encounter any similar problems and complications. Despite the mentioned possible causes of reoperation, only 5 late cardiac reoperations (10%) were performed for our patients, none of which were related to the Konno–Rastan procedure.

In the present study, re-exploration for bleeding was carried out for 4 cases (8%). The re-exploration rates in the literature ranged from 0% to 18.8% [15, 16]. In terms of bleeding, a study by Misbach *et al.* [17] concluded that covering the Dacron patch of the aorta with a pericardial patch reduced the risk of postoperative bleeding at the Dacron patch site.

In our study, mechanical prosthetic valve prothesis were used for all patients, except for 2 cases with biological valves on patientś request.

The use of a pulmonary autograft in aortic position after anterior aortoventriculoplasty, followed by homograft placement for the right ventricular outflow tract (Ross–Konno procedure), has been advocated and suggested as the procedure of choice for infants and children with small annulus in some studies [18, 19]. Low thrombogenicity, low risk of infection, excellent haemodynamics and even growth potential with long-term durability have been attributed to this procedure. Regardless of these advantages, the ultimate need for reoperation, either for the pulmonary autograft or for the homograft, being a more demanding procedure [12, 20]. In our centre, we perform the Ross–Konno procedure mainly for infants and younger children with a small aortic annulus diameter (<8–10 mm).

So far, various techniques have been introduced for aortic root enlargement, such as posterior root enlargement procedures, including the Nicks and Manouguian techniques, introduced by Nicks *et al.* [21] and Manouguian and Seybold-Epting [22] in 1970 and 1979, respectively. Although they are simpler procedures, root enlargement is limited to 1 or 2 sizes. Therefore, they are not suitable, especially for children, when the gap between the actual and the desired size is remarkable. In this regard, Yang *et al.* [*23*] recently reported posterior aortic root enlargement with a Y incision and a rectangular patch [24]. Although this procedure may enlarge the annulus by 2–5 sizes, it does not relieve the subvalvar component in patients with concomitant LVOT stenosis; also, its feasibility in the paediatric age group is unclear. On the other hand, the Konno–Rastan procedure may result in the most significant root enlargement [24] and resolve subvalvular and supravalvular stenosis, which are combined with the valvar component in many patients with small annuls.

The choice between different procedures depends on the patient's condition and the surgeon's experience and comfort, while also taking into consideration the advantages and disadvantages of each technique.

According to Table 4, although the major in-hospital complications were more common among patients with a valve diameter of <15 mm, their late complications were not significantly higher.

### Limitations

Although a sample size of 48 seems adequate for long-term follow-up, the results can be more applicable and generalizable if larger prospective, multicentre cohort studies are conducted. Also, the present results are only related to the Konno–Rastan method. Therefore, comparative studies, especially with a long-term follow-up, can be performed to compare this technique with other techniques for LVOT obstruction, such as the Ross-Konno procedure or posterior annulus enlargement techniques.

## CONCLUSION

Konno–Rastan surgery appears to be safe and highly effective for aortic root enlargement in children and adults.

There was no significant difference between patients with aortic annulus diameters <15 and >15 mm regarding late major complications. Although, the major in-hospital complications were more prevalent among patients with a valve diameter of <15 mm, a smaller annulus diameter may not preclude this procedure as the overall outcome is quite acceptable.

## CONSENT FOR PUBLICATION

This article does not contain any personal data, and the consent for publication is applied.

## Data Availability

All relevant data are within the manuscript and its Supporting Information files.

## ABBREVIATIONS

LVOT: Left ventricular outflow tract
RCA: Right coronary artery
PPM: Prothesis–patient mismatch
